# Analysis of Semen Proteomic Differences Among Three Genotypes of FecB Rams in Duolang Sheep

**DOI:** 10.3390/genes16101226

**Published:** 2025-10-16

**Authors:** Yanlong Zhang, Zhigang Niu, Jiabao Yan, Yang Chen, Zhengfen Xue, Jie Xu, Yifan Ma, Hongcai Shi

**Affiliations:** 1 College of Animal Science and Technology, Xinjiang Agricultural University, Urumqi 830052, China; 15962887647@126.com (Y.Z.);; 2 Xinjiang Key Laboratory of Animal Biotechnology, Key Laboratory of Herbivorous Animal Genetics, Breeding and Reproduction, Ministry of Agriculture and Rural Affairs, Institute of Biotechnology, Xinjiang Academy of Animal Sciences, Urumqi 830011, China; 3 College of Animal Science and Technology, Shihezi University, Shihezi 832061, China

**Keywords:** FecB, Duolang sheep, semen, proteomics

## Abstract

Backgrouds: To explore the differences in semen proteins among rams of three *FecB* genotypes (++, B+, and BB) in Duolang sheep. Methods:  This study employed DIA quantitative proteomics technology to identify semen proteins from four wild-type (Group A), two heterozygous (Group B), and three homozygous (Group C) rams. Results: Compared with the ++ genotype, the differentially expressed proteins (DEPs) in the semen of B+ genotype rams are significantly involved in the biological process of innate immune response and are significantly enriched in the oxidative phosphorylation pathway in KEGG analysis. From a biological perspective, the innate immune response may affect the immune health of Duolang sheep, while oxidative phosphorylation influences energy metabolism, which in turn impacts reproductive performance. Compared with the BB genotype, the DEPs in the semen of B+ genotype rams participate in biological processes such as protein phosphorylation and protein hydrolysis during cellular protein catabolism. These DEPs are also significantly enriched in pathways related to Parkinson’s disease and non-alcoholic fatty liver disease in KEGG analysis. These differences may affect the cellular metabolism and physiological functions of Duolang sheep, thereby being associated with their reproductive performance. Compared with the ++ genotype, the DEPs in the semen of BB genotype rams exhibit differences in molecular function, cellular component, KEGG pathway, domain function, and subcellular localization. For instance, they are involved in threonine-type endopeptidase activity and associated with pathways like Alzheimer’s disease and retrograde endocannabinoid signaling. Conclusions: These differences may have potential impacts on the physiology and reproductive performance of Duolang sheep.

## 1. Introduction

The sheep *FecB* gene, also known as the *BMPR1B* (Bonemorphogeneticproteinreceptortype-1B) gene, is an acronym for the English terms fecundity and booroola [1]. The name is based on the Nomenclature Committee on Sheep and Goat Genetics because of a mutation in the gene’s coding region, A764G, which can alter the trait of the number of lambs produced by Booroola Merino sheep. *FecB* manifests as three different genotypes: wild-type (++), heterozygous (B+), and pure breeds (BB). Heterozygotes (B+) can increase lambing by 0.9~1.2 lambs, while purebreds (BB) can increase lambing by 1.1~1.7 lambs [2]. Notably, the identification of the *FecB* gene and its mutations in the domestic sheep breeds Dolang and the successful breeding of three genotypes of rams and ewes do not match the performance of Booroola Merino in terms of the number of lambs produced and the distinctive phenotypes [3]. This phenomenon is accompanied by an increase in the number of abortions, weak lambs, and deaths. High-quality ram semen is the most crucial and essential component of artificial insemination to achieve optimal fertility and productive performance in rams. Sperm proteins and seminal plasma proteins are the major proteins found in semen. They are important for fertilization and semen viability and can both directly affect the molecular mechanisms and biological pathways that control sperm activity. Semen proteins can thus be employed as potential indicators for sperm dysfunction screening and semen quality assessment [4]. Proteomic analysis of sperm and seminal plasma protein profiles can reveal the intricate relationships between proteins and sperm function.

In this study, through proteomic analysis of semen from Duolang sheep rams with three FecB genotypes, this study aims to understand their impacts on reproductive performance, as well as the individual metabolism and physiological functions of rams, and to identify the potential effects on Duolang sheep and their reproduction.

## 2. Materials and Methods

### 2.1. Experimental Site and Test Animals

The experiment has passed the ethical review of the Science and Technology Ethics Committee of the Xinjiang Uygur Autonomous Region Academy of Animal Husbandry Sciences, with the ethical review number 202408012. Nine *FecB*-genotype rams, consisting of four wild-type (++) rams (Group A), two heterozygous (B+) rams (Group B), and three homozygous (BB) rams (Group C), were bred at the National Breeding Farm of Wuzheng Green Agriculture Co., Ltd. in Kashgar, Xinjiang, China. The rams were between the ages of two and three years and had a weight of roughly 120 kg. Besides the daily baseline diet, each ram was given one egg per day, supplemented with alfalfa hay (2 kg/d) and fresh carrots (1 kg/d), and taught to run 5 km two weeks before the experiment.

### 2.2. Equipment, Drugs, and Reagents Used

Experimental Instruments: Refrigerator, constant temperature water bath, microscope, frozen semen programmed cooling instrument, freezer, liquid nitrogen tank. Experimental Materials: Artificial vagina, semen collection cup, 5 mL vacuum test tube, 0.25 mL straw, 1 mL and 5 mL syringes, gauze, etc. Main Drugs and Reagents: Concentrated frozen semen diluent for sheep (purchased from Xinbo Biotechnology), distilled water, Vaseline, glycerol, absolute ethanol.

### 2.3. Semen Collection and Dilution Equilibration

Semen was collected from rams using the artificial vagina method. An estrous ewe was selected as the teaser, which was secured in a holding rack. The artificial vagina was preheated with warm water, and water was poured into it to occupy 1/3–1/2 of the volume between the outer and inner shells (i.e., when the artificial vagina was held upright, the water level reached the water filling hole). Vaseline was applied to the penis entry end for lubrication, and a semen collection cup was attached to the other end, forming a triangular shape at the Vaseline-coated end. After preparing the artificial vagina, it was placed beside the secured estrous ewe to wait. Semen collection was performed when the ram mounted the ewe and extended its genitalia. A drop of the collected semen was first placed on a glass slide, and its quality was observed with the naked eye under a microscope. Semen that showed cloudy rolling, milky white color, no abnormal odor, and a motility of over 90% was selected. The semen was diluted using the two-step dilution method: based on the semen density, it was first diluted 1–2 times with concentrated frozen semen diluent for sheep (preheated in a 37 °C water bath in advance). The diluted semen was then placed in a 4 °C refrigerator for 2 h of equilibration. After equilibration, an equal volume of freezing solution (at 4 °C) was added to dilute the original semen by 2–4 times, followed by another 2 h of equilibration in a 4 °C refrigerator.

### 2.4. Preparation of Straw Frozen Semen

The equilibrated semen was taken out of the refrigerator, gently shaken, and then aspirated into 0.25 mL straws, which were sealed with sealing powder. After sealing, the straws containing semen were evenly placed on a straw rack and equilibrated for approximately 5 min. After equilibration, the straw rack was quickly placed into a large container pre-filled with liquid nitrogen for fumigation. During this process, the distance between the straws and the liquid nitrogen surface was maintained at 0.8–1 cm. The container lid was closed, and fumigation was conducted for about 5 min. After fumigation on the liquid nitrogen surface, all straws were placed in a gauze bag, immersed in liquid nitrogen, and left for 10–30 min to complete the preparation of straw frozen semen. A 0.5 mL sample from each frozen semen batch was sent to Beijing Novogene Technology Co., Ltd. for Astral DIA quantitative proteomics analysis in Beijing, China.

### 2.5. Data Analysis

#### 2.5.1. Protein Identification and Quantification

The DIA-NN library search program was used to search and evaluate the raw files based on the UniProt protein database. The parameters for the library search were as follows: Up to two missed cut sites were permitted, the immobilization modification was the alkylation modification of cysteine, the loss of methionine at the N-terminal end was a variable modification, and the mass variation in precursor and fragment ions was automatically detected and rectified. The DIA-NN program further limited the search results to increase the quality of the analysis findings. Only proteins with PG.Q.Value < 0.01 (FC < * [fold change, FC]) and peptides with Global. Q.Value < 0.01 were kept. Proteins with a significant difference in quantification between the two groups (*p* < 0.05, |log2FC| > * (FC > * or FC < * [fold change, FC])) were classified as differentially expressed proteins (DEP). Raw data were deconvoluted, and species libraries were searched using the DIA-NN software (Direct DIA (Basic Version)) for Blood-Plus (full platform) [5].

#### 2.5.2. Functional Analysis of Proteins and DEP

The Interproscan software (5.72-103.0), which includes the Pfam, PRINTS, ProDom, SMART, ProSite, and PANTHER databases, was used to functionally annotate GO and IPR. COG and KEGG were used to perform functional protein family and pathway analysis of the identified proteins. Volcano plot analysis was done to identify significant DEPs, clustering heatmap analysis was employed to visualize the expression patterns, and pathway enrichment analysis using GO, IPR, and KEGG was carried out to understand the functional significance of the DEPs. The STRING DB program (http://STRING.embl.de/, accessed on 18 June 2025) was used to predict potential protein–protein interactions.

## 3. Results

### 3.1. Overview of the Number of Proteins Identified by DIA

**Figure 1:**
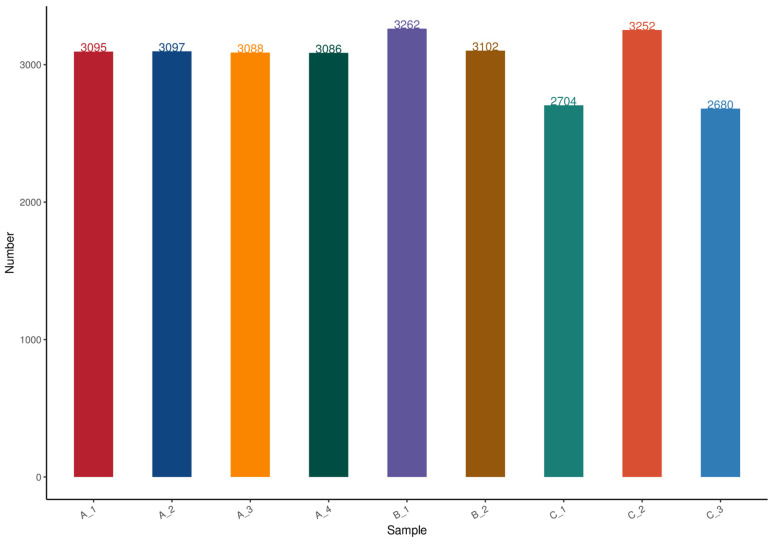
Overview of sample protein identification. The horizontal coordinate is the sample name, while the vertical coordinate is the number of proteins.

The DIA data were imported into the DIA-NN program to achieve the simultaneous characterization and quantification of peptides. The ion-pair chromatographic peaks were retrieved, and sub-ion matching and peak area calculations were carried out. The nine samples contained 3095, 3097, 3089, 3086, 3262, 3102, 2704, 3252, and 2680 proteins, respectively (Figure 1).

### 3.2. Functional Annotation of Proteins

**Figure 2:**
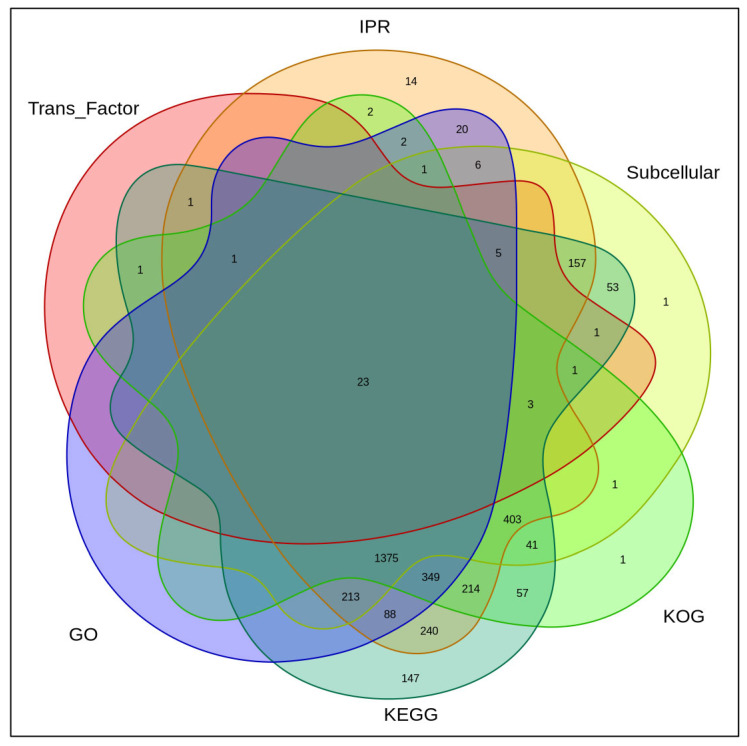
Functional annotation results. Each ‘circle’ in the figure represents the annotation results of one database. The overlapping part represents the proteins jointly annotated by multiple databases, while the non-overlapping part represents the proteins individually annotated by the corresponding databases.

GO, KEGG, KOG, Subcellular (structural domain), IPR (subcellular localization), and Tran_Factor jointly annotated 23 proteins., KEGG alone annotated 147 proteins, KOG alone annotated 1 protein, Subcellular (structural domain), IPR (subcellular localization), and Tran_Factor (transcription factor) jointly annotated 23 proteins, Subcellular (structural domain)KOG alone annotated 1 protein, and while IPR alone annotated 14 proteins, as shown in Figure 2. IPR alone, 14 proteins, and subcellular alone, 1 protein.

#### 3.2.1. Protein GO Functional Annotation

**Figure 3:**
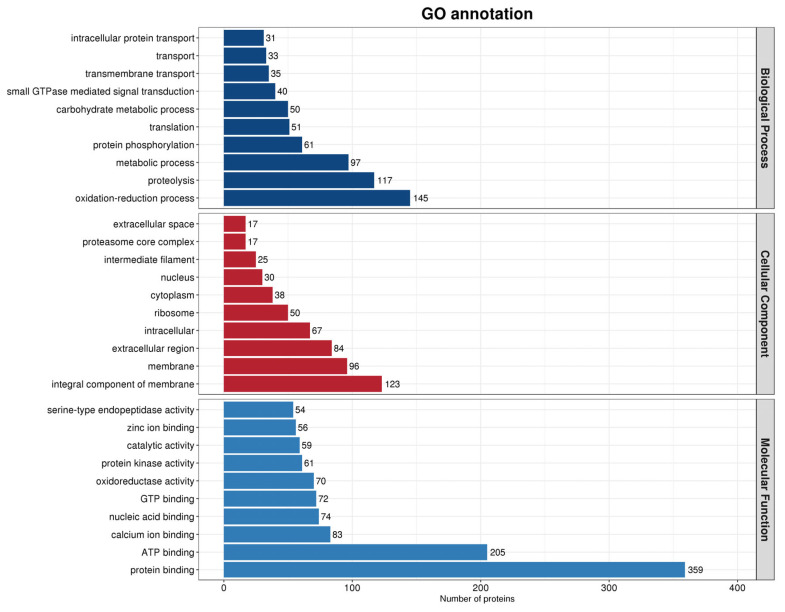
Histogram of all GO annotation results. The horizontal coordinates represent the number of proteins, while the vertical coordinates represent the GO entries to which the proteins are annotated.

The GO annotation results of every protein in the samples were grouped into three primary categories: Molecular function, cellular component, and biological process (Figure 3). The molecular function primarily describes the individual functions of the gene and its product, such as the ability to bind carbohydrates or the activity of ATP to hydrolyze enzymes. The cellular component primarily describes the subcellular structure, location, and macromolecular complexes, such as nucleolus, telomere, and recognition initiation complexes. The biological process primarily describes the function of the gene and its product. Only the top 10 findings in each broad category were included in the figure (Figure 3) because of the vast number of entries in the GO annotation results. Notably, there were a lot more proteins involved in molecular processes than those involved in biological processes and cellular components. The molecular functions of proteins are essential for maintaining regular cellular life activities. Variations in protein levels impact the cell’s ability to operate normally, which subsequently has an impact on the sperm flagellum’s normal oscillation. Notably, the number of proteins involved in biological processes was closer to the number of proteins involved in cellular components. Changes in the composition of cellular fractions potentially affect the normal morphology and activity of the cells. In contrast, biological processes are involved in the normal motility of spermatozoa and jointly regulate sperm viability through the interactions between the various components. Sperm abnormality rates and motility are thus potentially correlated with the proteins that contribute to the cellular components of semen.

#### 3.2.2. Functional Annotations

**Figure 4:**
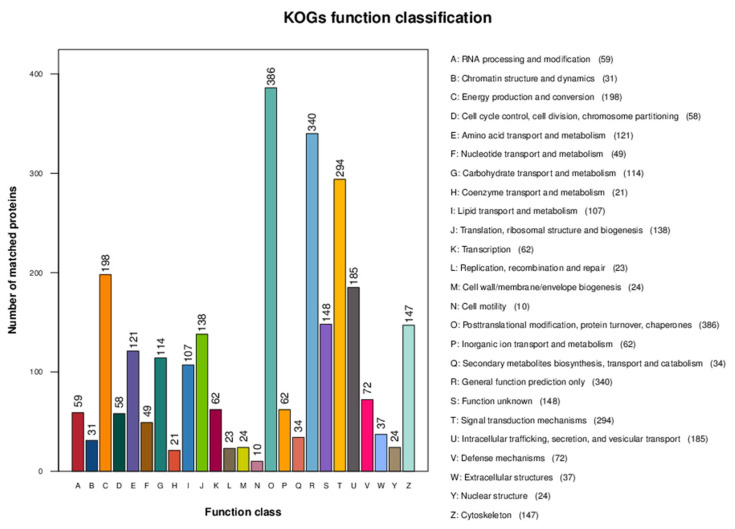
Histogram of all COG annotation results. The horizontal coordinate is the functional classification of the annotation, while the vertical coordinate is the number of proteins annotated to the corresponding function.

Cluster of Orthologous Groups of proteins (COG) presents the classification results of all proteins (Figure 4). Notably, the frequency of posttranslational modification, protein turnover, and chaperones is significantly higher than that of other categories.

#### 3.2.3. Functional Annotation of All KEGG

**Figure 5:**
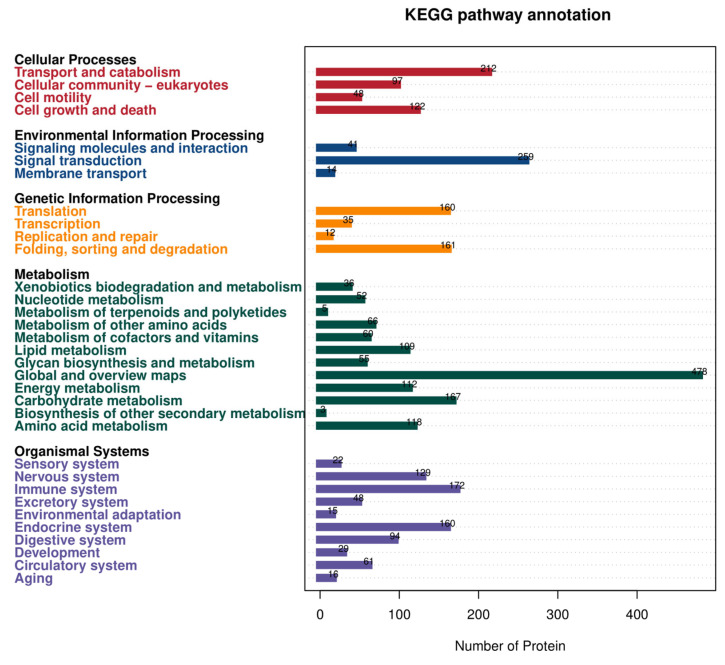
Histogram of KEGG annotation results. Horizontal coordinates represent the number of proteins, while the vertical coordinates represent the KEGG entries to which the proteins are annotated.

Figure 5 shows the categories of KEGG annotation findings for all proteins. Notably, 33 biological pathways were involved, with most of these pathways being engaged in five different kinds of biological activities. The proteins had a wide range of biological functions in cell physiology, environmental information processing, genetic information processing, metabolism, and tissue systems. The number of proteins involved in metabolism and tissue systems was substantially more than that involved in cell physiology, environmental information processing, and genetic information processing. There were 478 relevant proteins in global and overview maps, which was much more than the number of proteins in other pathways. These proteins revealed the architecture of the whole metabolic network, providing a more macroscopic picture of the inherent links between the main metabolic pathways in the cell rather than concentrating on the specifics of a given biochemical activity. The proteins were essential to spermatozoa’s ability to sustain regular metabolic processes, giving them a strong metabolic base that ensures their regular physiological function.

#### 3.2.4. Annotation of All Structural Domains

**Figure 6:**
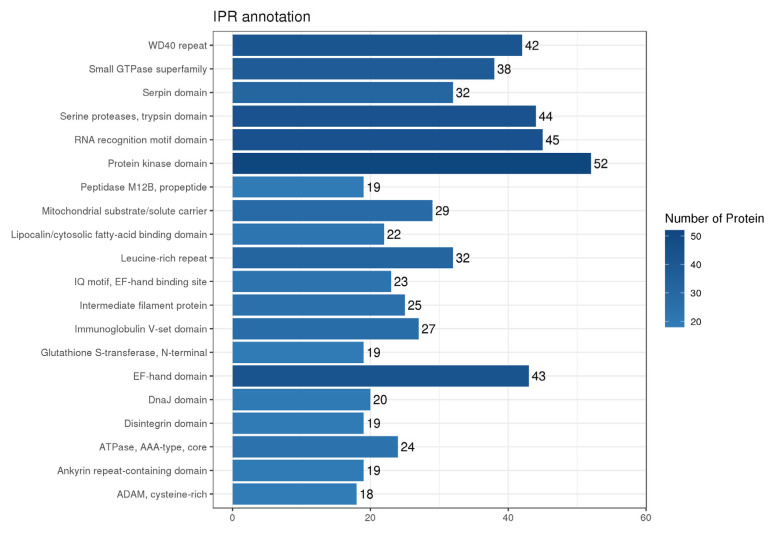
Histogram of structural domain annotation results. Horizontal coordinates represent the number of proteins, while the vertical coordinates represent the IPR entries to which the proteins are annotated.

The structural domains of proteins are essential to the biological functions of proteins because they are the fundamental building blocks of protein structure, function, and evolution. The structural domains of the identified proteins were thoroughly investigated to accurately understand the biological roles of proteins and their lengthy evolutionary history. Figure 6 displays the categorization of the structural domain annotation outcomes for every protein. Of note, 20 distinct structural domains were identified. There were 52 domains of protein kinase, 45 domains of RNA recognition motif, 44 domains of serine protease and trypsin, and Calcium ion. In the same line, 42 WD40 repeat proteins and 43 high-affinity structural domains unique to calcium-binding proteins were identified. These structural domain types were substantially more numerous than the others and could thus be potentially important for the execution and interactions of protein functions.

#### 3.2.5. Annotation of All Subcellular Localization Information

**Figure 7:**
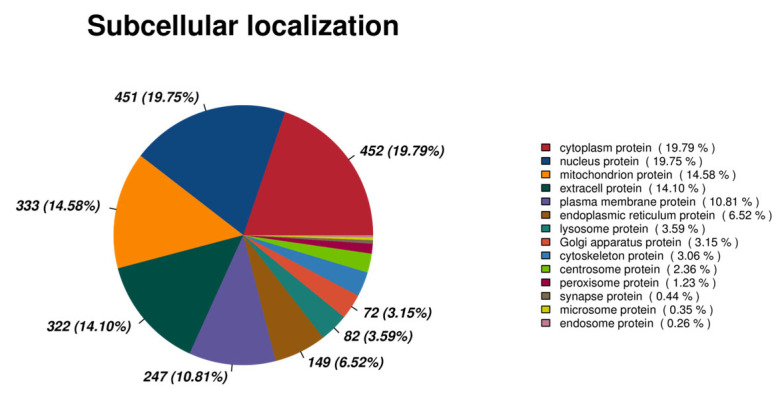
Pie chart of subcellular localization annotation results.

Cells are highly complex and well-organized structures. Numerous distinct organelles or cellular areas, including the nucleus, Golgi apparatus, endoplasmic reticulum, mitochondria, cytoplasm, and cell membrane, can be separated inside the cell based on variations in their spatial distribution features and activities. Notably, proteins are precisely delivered to certain organelles once synthesis is finished in the ribosomes because of the protein sorting signals they contain. Moreover, some proteins are localized in the cytoplasm or are released to the external environment. Noteworthy, proteins can only engage in the many functions of the cell and fulfill their assigned duties when they are carried to the appropriate location. A thorough understanding of the subcellular localization of proteins is thus crucial to deciphering the mechanisms behind an organism’s activities and the control of physiological processes. Herein, 14 subcellular localization data, which included 452 cytoplasmic proteins, 451 nucleus proteins, 333 mitochondrion proteins, and 333 extracellular proteins, were analyzed based on the annotation results of subcellular localization data for all proteins (Figure 7). Of note, there were significantly more extracellular proteins than obtained in the subcellular localization data. Cytoplasmic proteins play a supportive, nutritive, connective, and protective role. In contrast, nuclear proteins are primarily involved in various proteins, DNA synthesis enzymes, RNA transcription and enzyme processing. They are also involved in various regulatory roles of protein factors, among other things, which are critical to the formation of spermatozoa. Mitochondrial proteins can generate energy and participate in cell signaling, cellular signaling and disease relevance, and apoptosis and disease relevance, which are important for sperm vitality and sperm-egg binding. Extracellular proteins have structural support, signal transduction, material transportation, and special functions that protect the structure of the sperm and provide nutrients and signals.

#### 3.2.6. PCA

**Figure 8:**
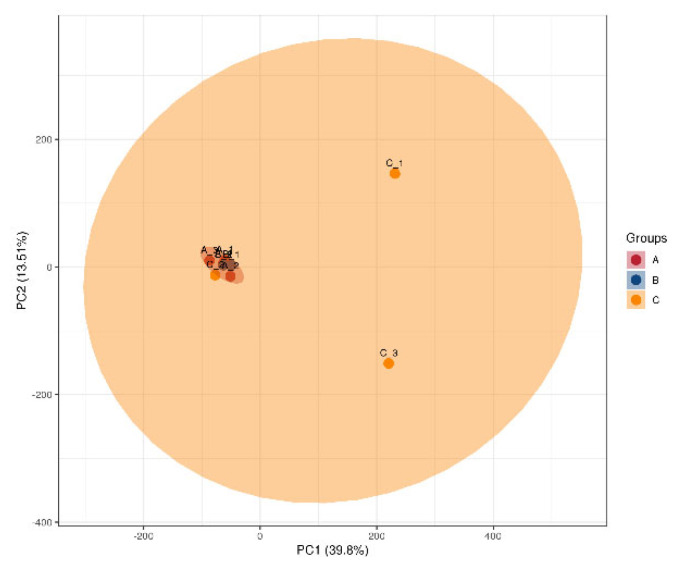
PCA of total samples. The horizontal and vertical coordinates PC1 and PC2 in the figure indicate the scores of the first and second ranked principal components, respectively. The scatter color indicates the experimental grouping of the sample.

Principal component analysis (PCA) is a traditional statistical technique that focuses on the capacity to manipulate a collection of connected variables. The set groups were subjected to PCA typing for analysis (Figure 8). Notably, the main component analysis graph of the entire sample revealed a significant difference in Group C’s proteome from that of Groups A and B. Group C also had significant differences within the group as well as overlaps between the protein components of Group C and those of Groups A and B.

**Figure 9:**
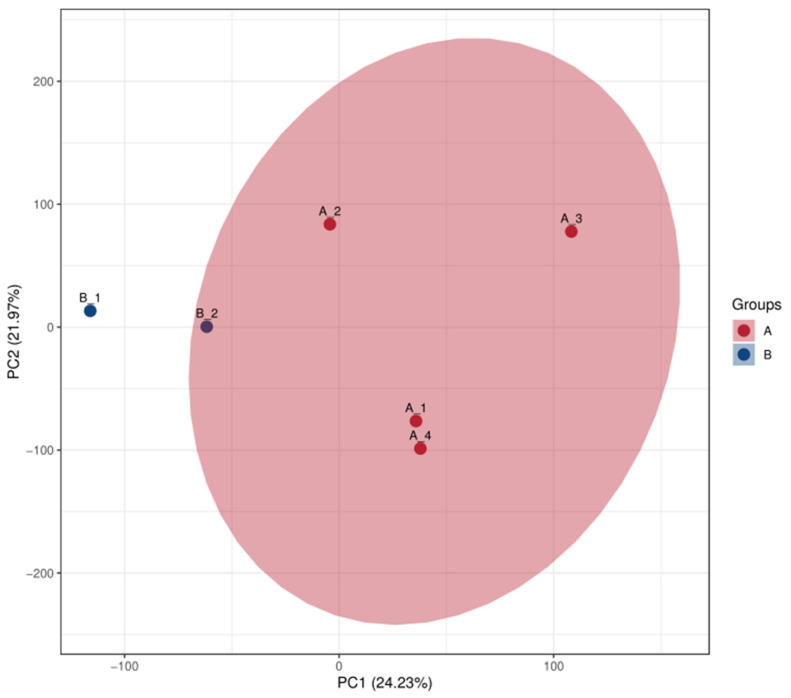

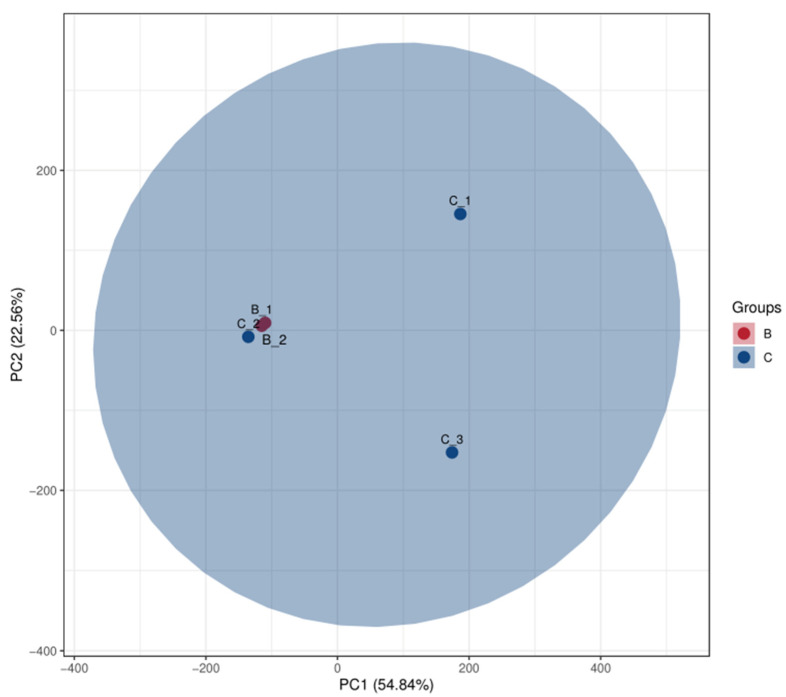

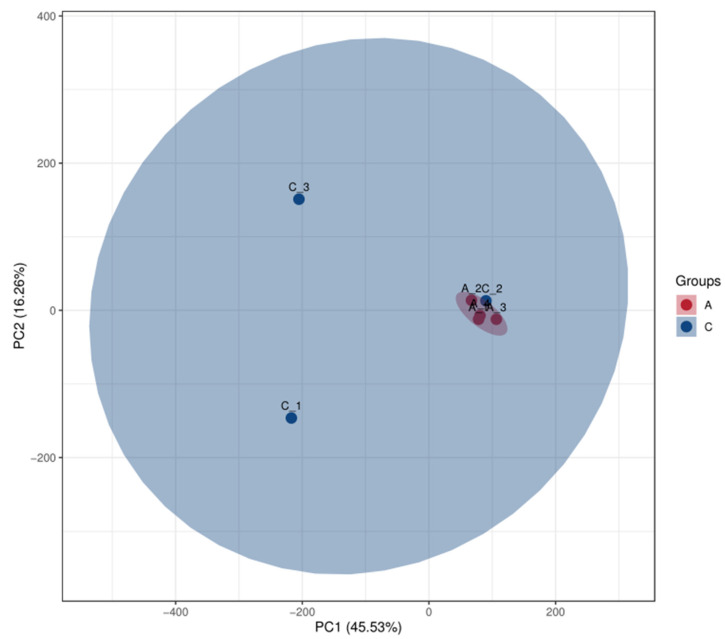
Comparison of PCA of pairs of samples.

The general distribution trend between the two sample groups was examined using PCA. The difference within group C was greater than that within group B. In contrast, the difference within group A is lower than that within group B (Figure 9). The samples in groups A and B were more widely distributed in the PCA, while the samples in groups B and C were more densely packed. Samples in group C were more widely distributed. PCA of groups A and C revealed that the samples in group A were densely packed while the samples in group C were scattered.

### 3.3. Screening for Differential Proteins

**Figure 10:**
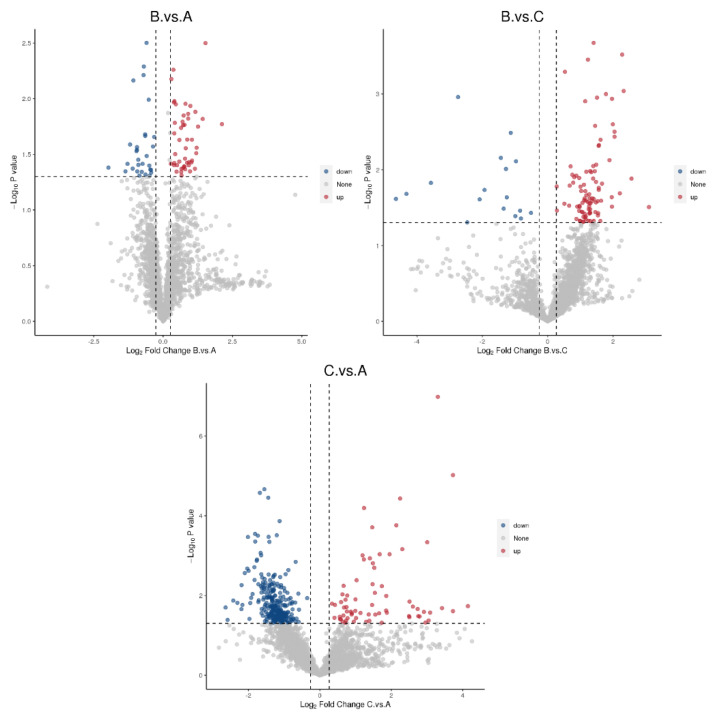
Volcano plots comparing the differentially expressed proteins between groups. Gray represents proteins with insignificant differences, red represents up-regulated proteins, and blue represents down-regulated proteins.

**Figure 11:**
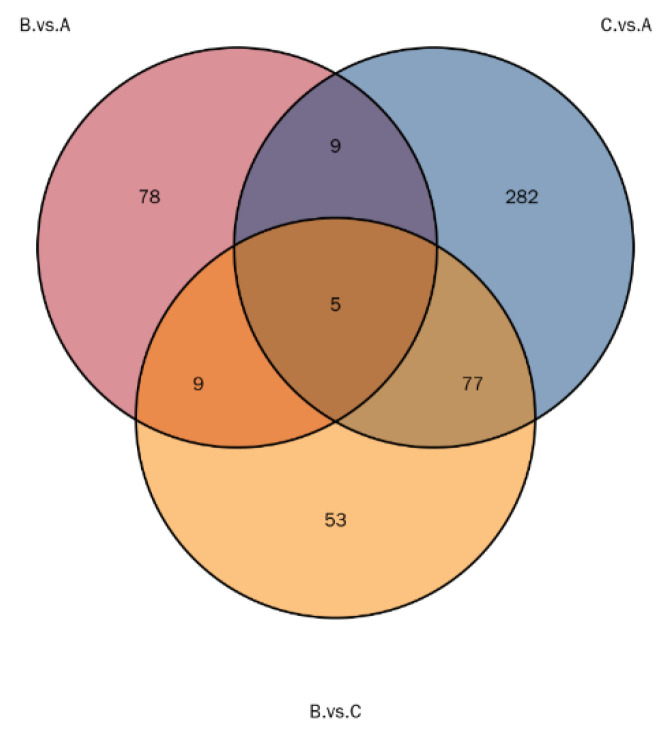
Wayne diagram of differential proteins. Each circle in the figure represents the differential protein results for one comparison combination. The overlap represents the differential proteins common to multiple comparison combinations, while the non-overlap represents differential proteins that only appear in one group.

Groups B and A yielded 2989 proteins, groups C and A yielded 2827 proteins, while groups B and C yielded 2771 proteins (Table 1). Notably, 35 proteins were down-regulated in the comparison of groups B and A, 292 proteins were down-regulated in the comparison of groups C and A, and 28 proteins were down-regulated in the comparison of groups B and C. In contrast, 116 proteins were upregulated in the comparison of groups B and C, 81 proteins were upregulated in the comparison of groups C and A, and 66 proteins were upregulated in the comparison of groups B and A. The upregulated proteins had a fold change (FC) > 1.2 and *p* value < 0.05, while the down-regulated proteins had a fold change (FC) < 0.83 and *p* value < 0.05. A volcano diagram was created after a *T*-test was used to analyze the significantly different proteins between the two groups (Figure 10). The multiplicity of differences for each protein was calculated as the logarithm of the base of 2. In contrast, the *p* value was calculated as the negative of the logarithm of the base of 10. Multiple combinations of differentially expressed proteins were compared to generate a Wayne diagram (Figure 11). The number of differentially expressed proteins shared by the BA and CA groups was 9, the CA and BC groups were 77, and the B and BC groups were 1000. There were five common differential proteins and 77 common differential proteins across the three groups and nine common differential proteins between the BC and BA groups.

#### 3.3.1. Enrichment Analysis of Differential Proteins

**Figure 12:**
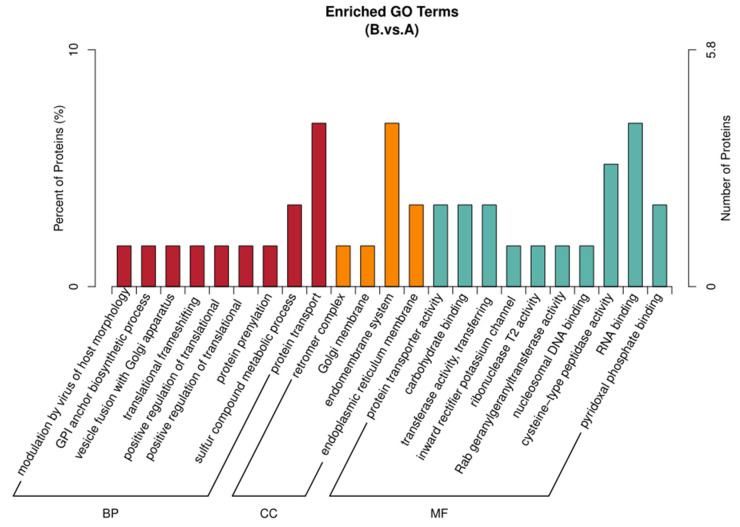

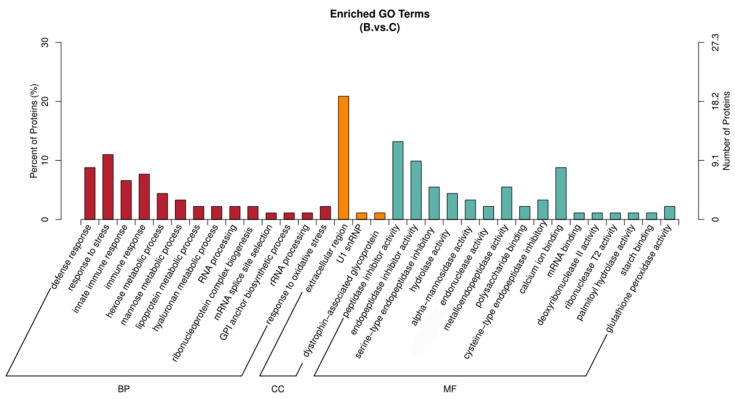

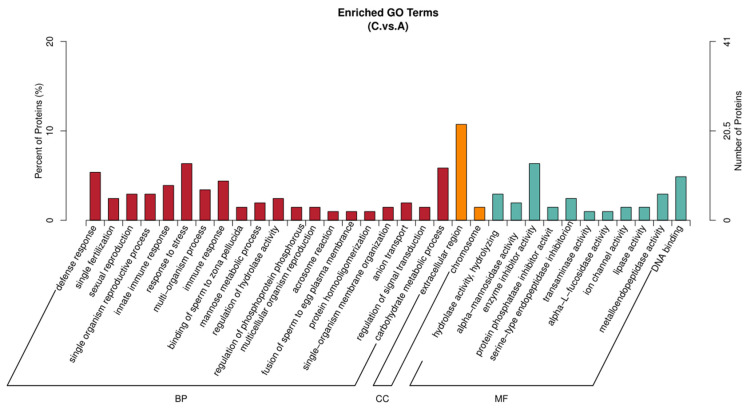
Histogram showing the GO enrichment results of differential proteins for the various comparison group. The enrichment results are in three categories of up to 20 species each (*p* value < 0.05). The vertical coordinate on the left is x/n, while the vertical coordinate on the right is the number of differential proteins annotated.

Figure 12 shows the GO functional annotation analysis results of differential proteins identified by comparing two groups. The enrichment results were in three categories, and the differential proteins were expressed in all three biological functions. Group B had the most diverse types of differential proteins involved in molecular function (MF) compared to Group A, with a total of 10 molecular functions involved. The proteins were mainly enriched in RNA binding and catalytic enzyme activity, which are potentially involved in gene regulation and sperm formation. Differential proteins in biological processes (BP) were concentrated in nine processes but were mainly involved in protein transport and sulfur compound metabolism. The least differentially expressed proteins in the cellular components (CC) were mainly located in the cell membrane and endoplasmic reticulum membrane. Group B had the largest number of differentially expressed proteins involved in molecular function (MF) compared to group C, with a total of 16 molecular functions. These proteins were mainly involved in peptidase inhibitor activity, endopeptidase inhibitory activity, and calcium ion binding. These molecular functions are potentially involved in molecular functions that affect sperm oxidative stress regulate physiological metabolism, and gene expression. Differential proteins in biological processes (BP) were concentrated in 14 processes. The three main biological processes were stress response, defense response, and immune response. The least differentially expressed proteins in the cellular component (CC) were mainly located in the outer region of the cell membrane, which is a vital site for material and information exchange between cells and the external environment. There were 20 differentially expressed proteins involved in biological processes (BP) in group C compared to Group A. These proteins were mainly associated with defense response, stress response, carbohydrate metabolism, and immune response. These biological functions were potentially associated with indicators such as sperm motility and dynamic parameters. There were 11 differentially expressed proteins in molecular function (MF). They were mainly associated with enzyme inhibitory activity, DNA binding, metalloendopeptidase activity, and hydrogenase activity. The least differentially expressed proteins in cellular components (CC) were mainly located in the outer regions of the cell membrane and chromosomes.

#### 3.3.2. Comparison of KEGG Enrichment Analysis

**Figure 13:**
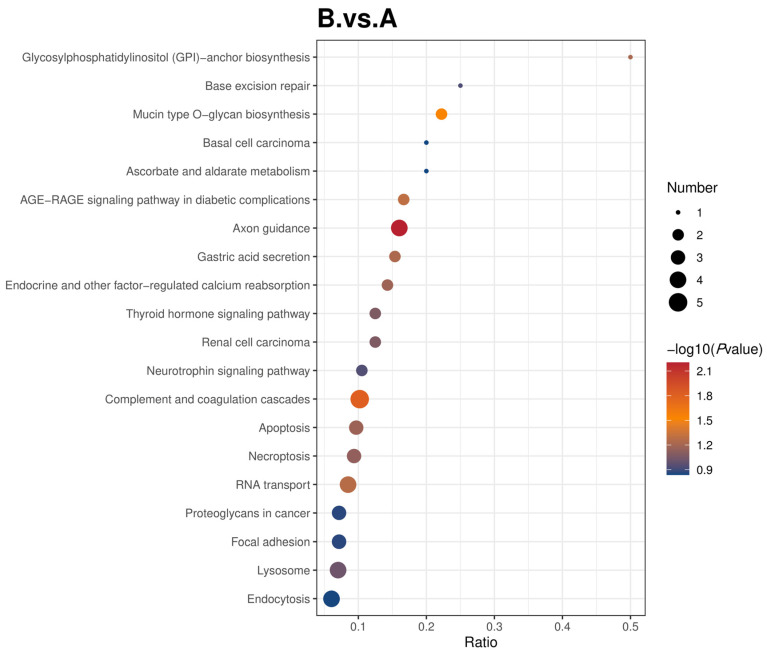

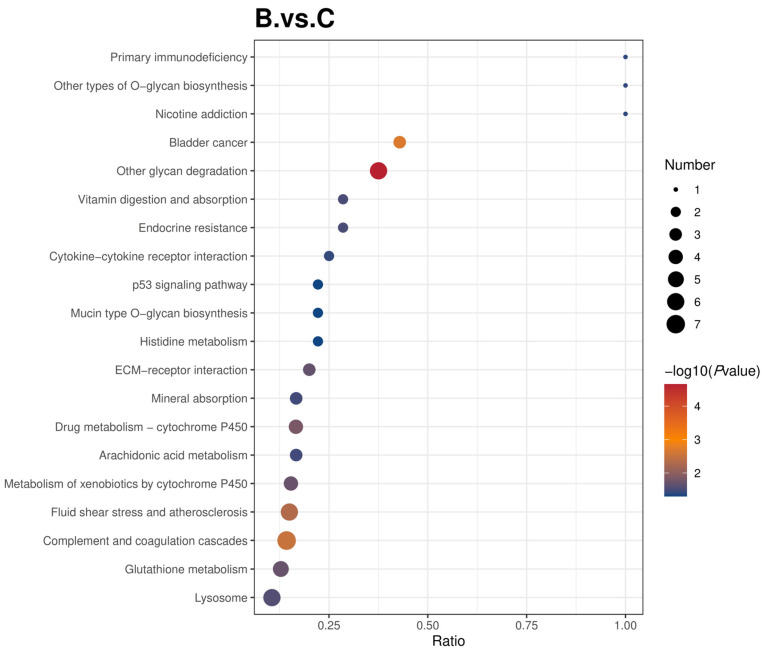

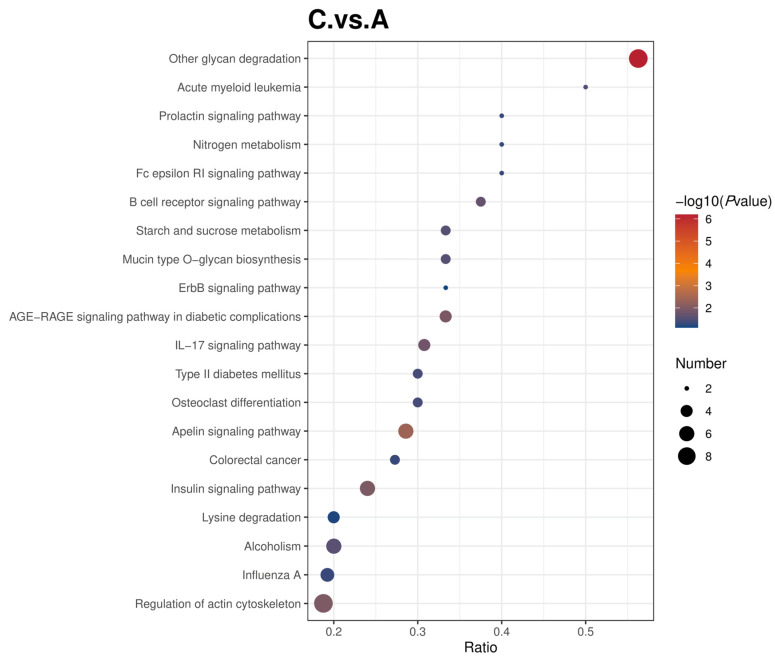
Bubble map showing KEGG enrichment analysis results for the different comparison groups. The horizontal coordinate in the graph is the ratio of the number of differential proteins in the corresponding pathway to the total number of proteins identified in that pathway. The larger the value, the higher the enrichment of differential proteins in that pathway and vice versa. The color of the dots represents the *p* value of the hypergeometric test. It ranges from blue to red; the redder the color, the smaller the *p* value, indicating that the test is more reliable and statistically significant. The size of the dots represents the number of differential proteins in the corresponding pathway, the larger the dot, the more differential proteins are in the pathway, and vice versa.

This study used the KEGG pathway as a unit to apply hypergeometric testing for enrichment analysis of the annotation results of differential proteins. The smaller the *p*-value, the greater the reliability of the test and vice versa. Figure 13 is a KEGG enrichment bubble plot of the various comparison groups. Glycosylphosphatidylinositol (GPI)—anchored protein biosynthesis was the pathway with the most significant enrichment level, while endocytosis had the least significant enrichment level among the 20 pathways enriched and analyzed in the comparison of group B and A. Primary immunodeficiency, other types of O-glycan biosynthesis, and nicotine addiction were the most significantly enriched pathways, while lysosome had the least significant enrichment level among the 20 pathways enriched and analyzed in the comparison of group B and C. Glycan degradation was the pathway with the most significant enrichment level, while the regulation of the animal cytoskeleton had the least significant enrichment level among the 20 pathways enriched and analyzed in the comparison of group C and A.

**Figure 14:**
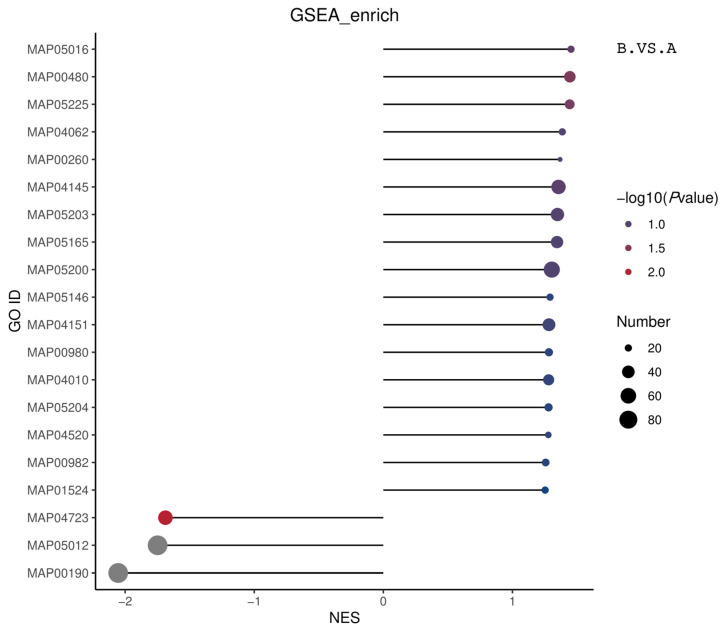

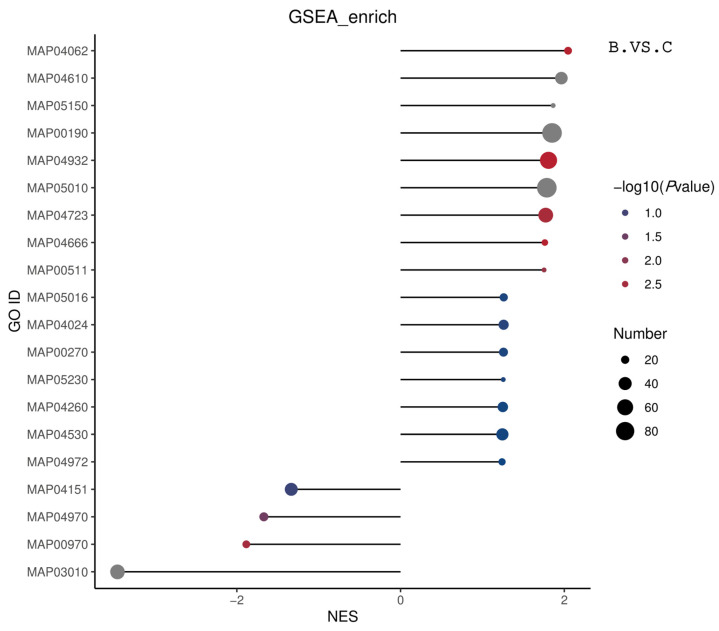

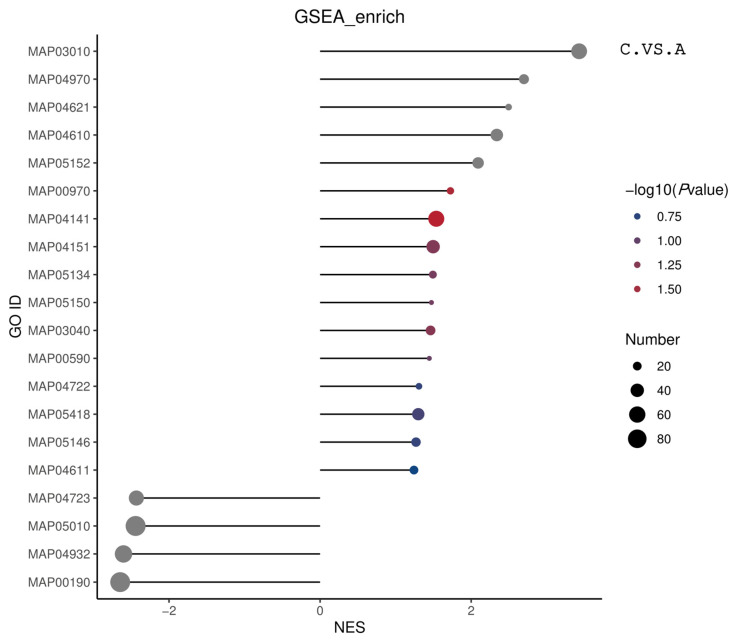
Matchstick plot showing the overall GSEA-KEGG ES enrichment results. The horizontal coordinate in the graph is the NES value. The larger the |NES| value, the higher the degree of differential protein enrichment in the entry and vice versa. The color of the dots represents −log10 (*p* value). The *p* value refers to the nominal *p* value in GSEA. The redder the color, the larger the −log10 (*p* value), while the smaller the *p* value, the larger the reliability of the test and, the more statistically significant the test is. The gray color denotes the pathways with a *p* value of 0. The size of the dots represents the number of differential proteins in the corresponding entry. The larger the dots, the higher the enrichment of differential proteins in the pathway and vice versa. The size of the dots represents the number of differential proteins in the corresponding entry. The larger the dots, the higher the number of differential proteins in the pathway and vice versa.

Table 2 outlines the GSEA-KEGG enrichment results. Group B was significantly enriched in the OXIDATIVE PHOSPHORYLATION pathway compared to Group A. The KEGG-ID: MAP05012 Parkinson’s disease and KEGG-ID: MAP04932 nonalcoholic fatty liver disease pathways were significantly enriched in group B compared to group C. The KEGG-ID: MAP05010 Alzheimer’s disease and KEGG-ID: MAP04723 retrograde endocannabinoid signaling pathways were significantly enriched in Group C compared to Group A. Figure 14 summarizes the GSEA-KEGG enrichment results by displaying the top 20 | NES | entries from BP, CC, and MF. All results have been displayed in cases where the entries are less than 20.

#### 3.3.3. Structural Domain Enrichment Analysis of Comparative Samples

**Figure 15:**
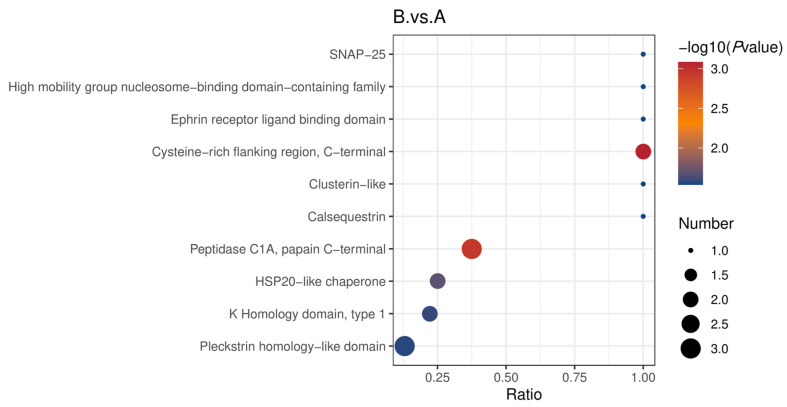

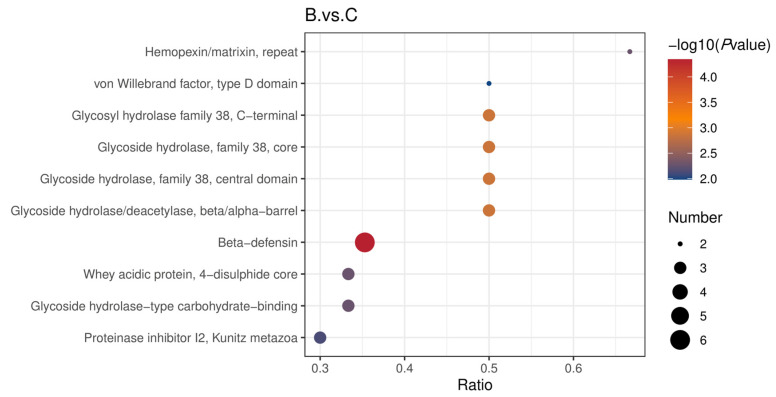

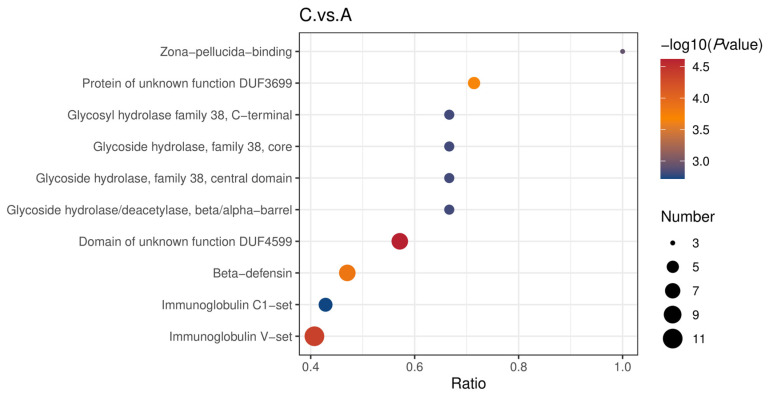
Enrichment bubble diagram of structural domains of the different comparison groups. The horizontal coordinate in the graph is the ratio of the number of differential proteins in the corresponding structural domain to the total number of proteins identified in that structural domain. The larger the value, the higher the enrichment of differential proteins in that structural domain and vice versa. The color of the dots represents the −log10 (*p* value) of the hypergeometric test. The redder the color, the larger the −log10 (*p* value), while the smaller the pvalue, the larger the reliability of the test and the more statistically significant it is. The size of the dots represents the number of differential proteins in the corresponding structural domains, the larger the dots. The higher the number of differential proteins in that structural domain.

Figure 15 displays the findings of structural domain enrichment of differentially expressed proteins. A total of 10 structural domains were enriched in the comparison of groups B and A. Pleckstrin homology-like domains, which primarily have multiple biological functions in the cell, particularly in the regulation of cell death and tumor suppression, were not significantly enriched. In contrast, a neurotransmitter-release related protein (SNAP-25), which helps regulate the fusion of the synaptic vesicle with the cell membrane, thereby releasing neurotransmitters, was the structural domain with the highest level of enrichment. The hemopexin/matrixin (REPEAT), which protects against oxidative stress damage and plays a role in the epithelial–mesenchymal transition, was the most significantly enriched domain among 10 domains that were enriched in the comparison of groups B and C. Zona-pellucida-binding was the most significantly enriched structural domain in the comparison of groups C and A. In contrast, the enrichment level of Kunitz metazoa (Proteinase inhibitor l2, Kunitz metazoa) was not significant, while the immunoglobulin V-set structural domain, which is primarily engaged in antigen binding, diversification, and information transmission, was not considerably enriched.

**Figure 16:**
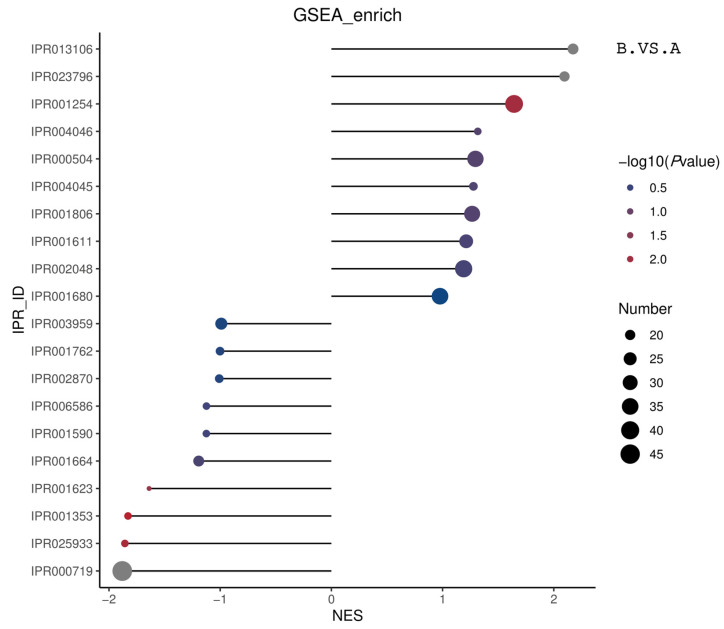

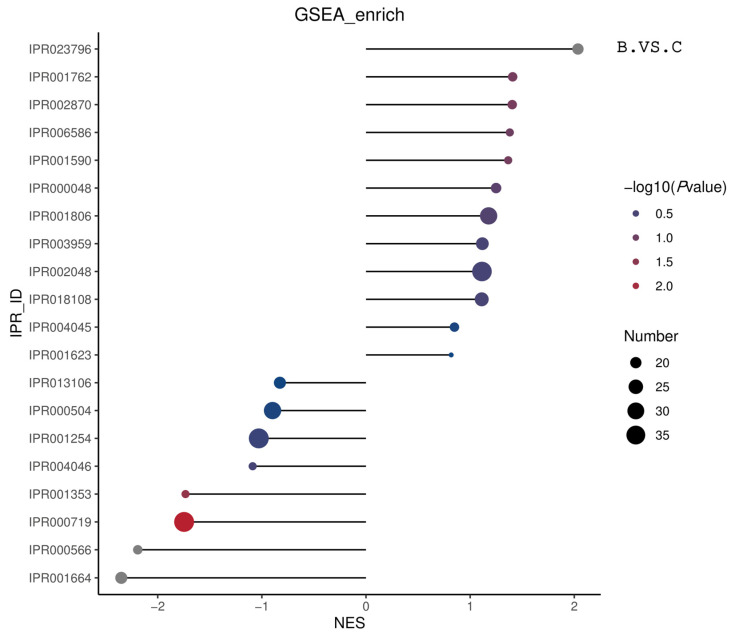

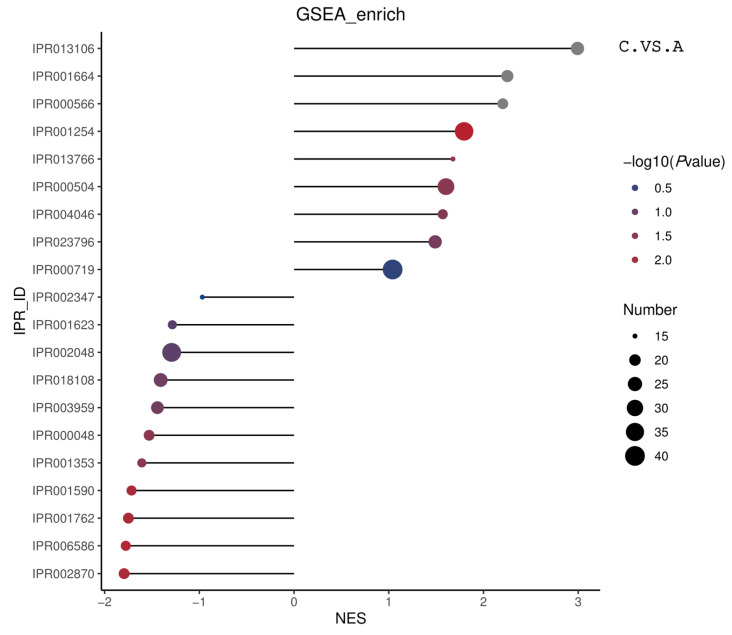
Matchstick diagram of the overall enrichment results of the GSEA-structural domain ES. The horizontal coordinate in the graph is the NES value. The larger the |NES| value, the higher the degree of differential protein enrichment in the entry. The color of the points represents −log10 (*p* value). *p* value refers to the nominal *p* value in GSEA. The redder the color, the larger the −log10 (*p* value), while the smaller the *p* value, the larger the reliability of the test and the more statistically significant the test is. The gray color represents the structural domains with a *p* value of 0. The size of the points represents the number of differential proteins in the corresponding entry. The larger the point, the higher the enrichment of differential proteins in that structural domain. The size of the dot represents the number of differential proteins in the corresponding entry. The larger the dot, the higher the number of differential proteins in the structural domain.

Table 3 outlines the GSEA domain enrichment results. There was a significant enrichment of the PROTEIN KINASE DOMAIN protein kinase region in the comparison of groups B and A. The functions of BETA-DEFENSIN and PROTEASOME, SUBUNIT ALPHA-BETA proteasomes were significantly enriched in the comparison of groups B and C. IPR_ID: IPR001623DNAJ domain function was significantly enriched in the comparison of groups C and A. Figure 16 summarizes the GSEA domain enrichment results by displaying the top 20 | NES | entries from BP, CC, and MF. All results are displayed in cases where the number of entries is less than 20.

#### 3.3.4. Subcellular Localization Analysis of Differential Proteins in the Different Comparison Groups

**Figure 17:**
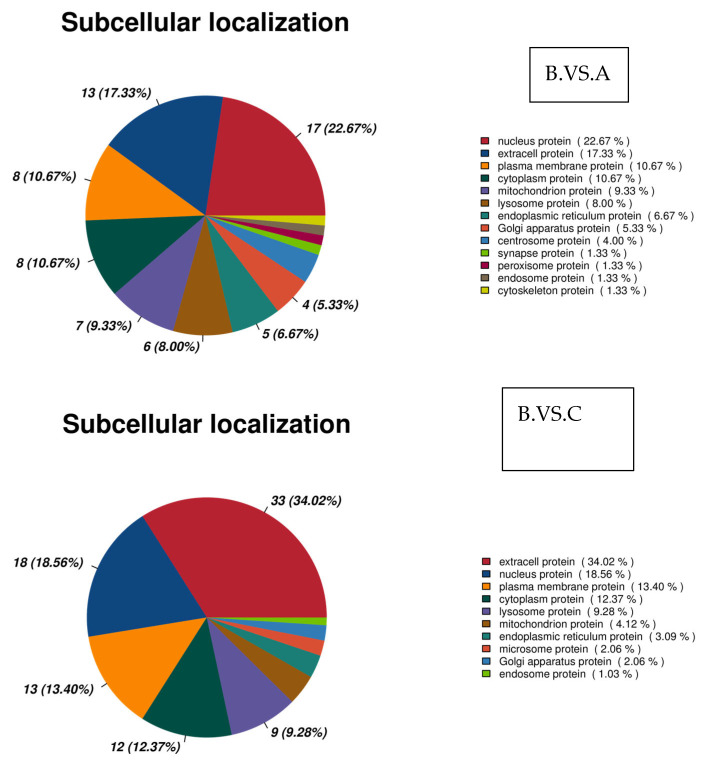

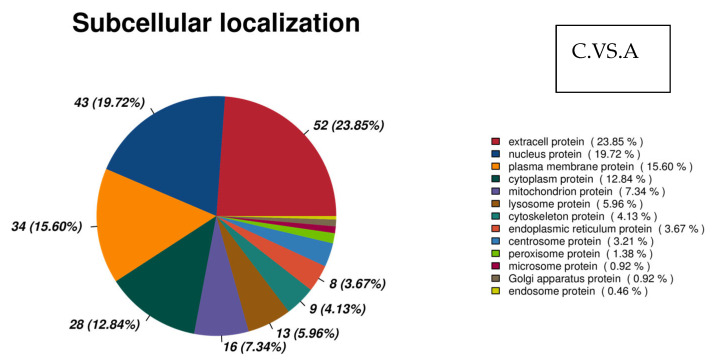
Analysis of subcellular localization of differential proteins.

The annotation results of subcellular localization information of proteins in different comparison groups were categorized (Figure 17). Thirteen subcellular localization items were annotated in the comparison of groups B and A. Notably, 13 extracellular protein items and 17 nucleus protein items were significantly higher than other subcellular localization information. Twenty-three extracellular proteins and eighteen nucleus proteins were significantly higher than other subcellular localization information in group B, which had ten subcellular localization information annotated overall. In group C, thirteen subcellular localization information were annotated overall, compared to fifty-two extracellular proteins and thirteen nucleus proteins in group A. Group C had a significantly higher amount of annotated subcellular localization information than group A. Among the 13 that were annotated, 52 were extracellular proteins, 43 were nucleus proteins, 34 were plasma membrane proteins, and 28 were cytoplasmic proteins.

**Figure 18:**
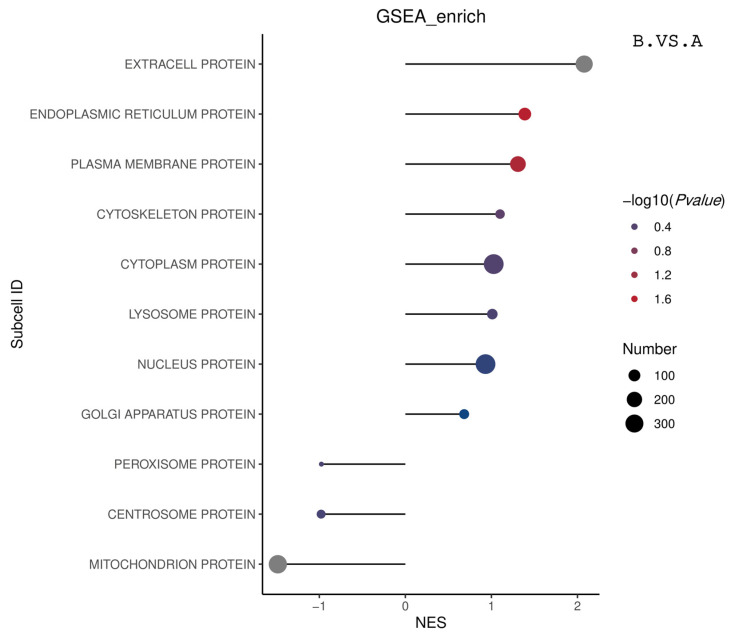

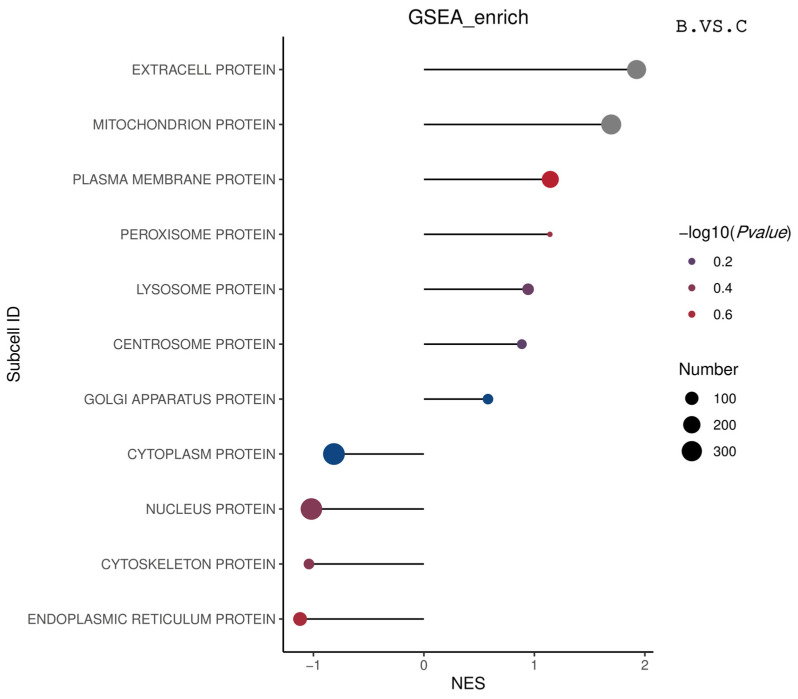

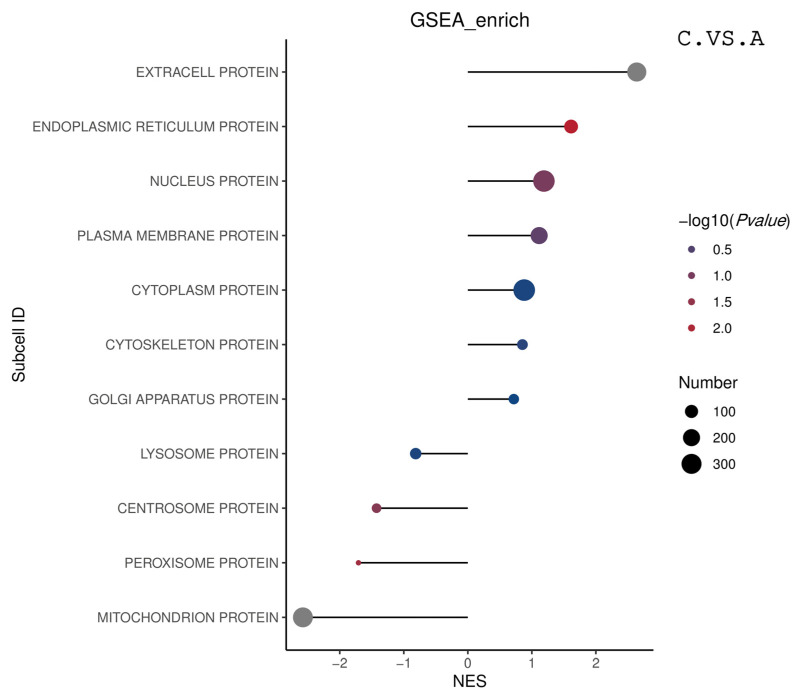
Matchstick plot of the overall GSEA-subcellular ES enrichment results. The horizontal coordinate in the graph is the NES value. The larger the |NES| value, the higher the degree of differential protein enrichment in the entry. The color of the dots represents −log10 (*p* value). The *p* value refers to the nominal *p* value in GSEA. The redder the color, the larger the −log10 (*p* value), while the smaller the *p* value, the larger the reliability of the test and the more statistically significant the test is. The gray color represents the subcellular entries with a *p* value of 0, the actual *p* value is extremely small and cannot be accurately obtained. The size of the dots represents the number of differential proteins in the corresponding entries, The larger the dots, the higher the number of differential proteins in the subcellular entries.

Table 4 outlines the GSEA subcellular enrichment results. The mitochondrial protein (MITOCHONDRION PROTOIN) localization information in Group B was significantly enriched compared to Group A. The localization information of extracellular and endoplasmic reticulum proteins in group C was significantly enriched compared to group A. Figure 18 summarizes the GSEA subcellular enrichment results by displaying the top 20 | NES | entries from BP, CC, and MF. All results are displayed in cases where the entries were less than 20.

#### 3.3.5. Differential Protein Interaction Analysis

**Figure 19:**
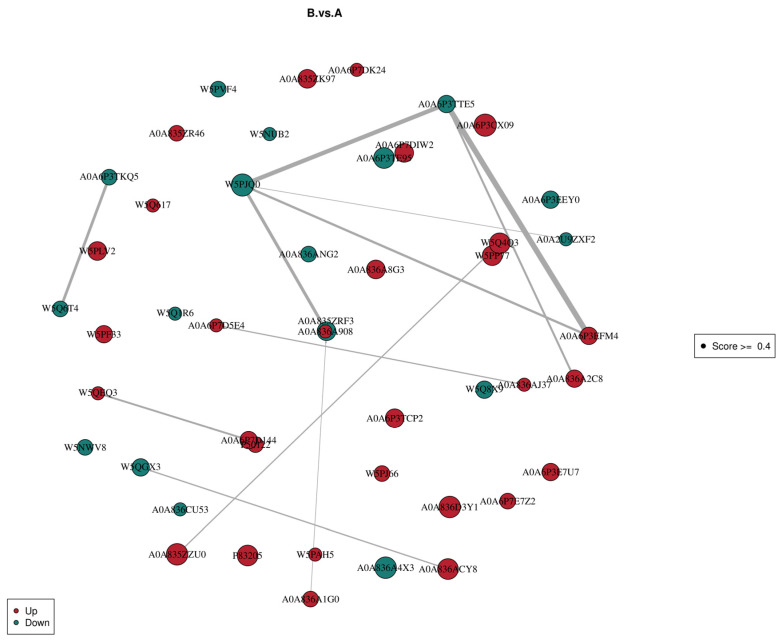

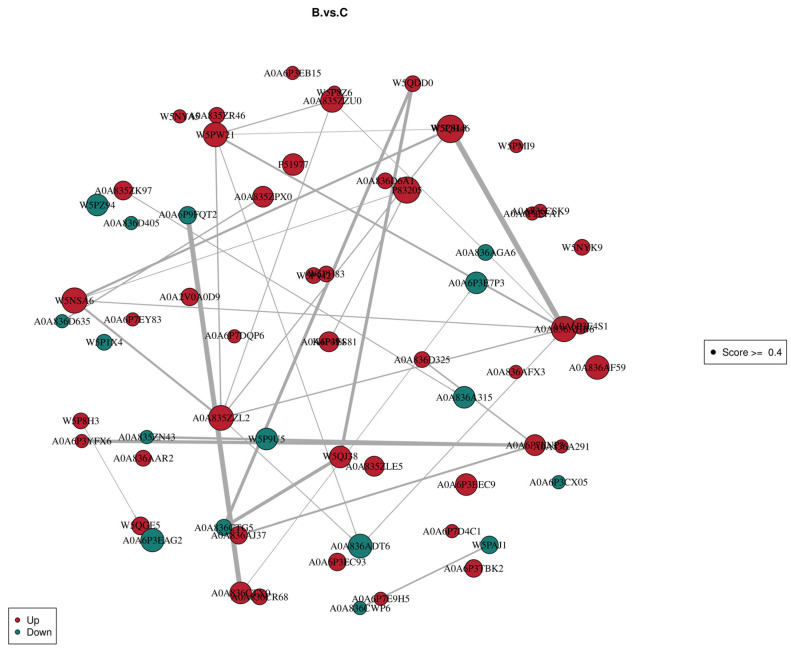

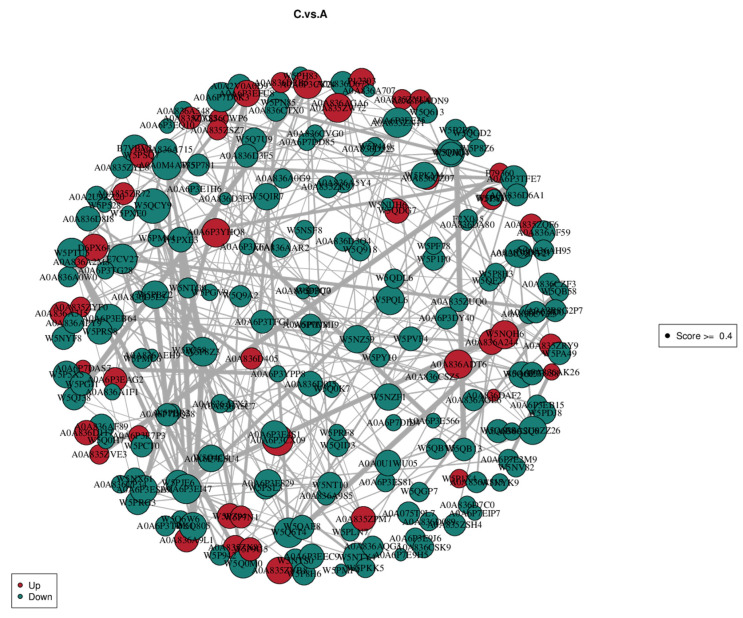
Protein interaction network diagram of the different comparison groups.

The differential expression analysis results and the interaction pairings in the StringDB protein interactions database were combined to effectively create differential protein interaction networks. Figure 19 shows the protein interaction networks of the top 50 differently expressed proteins in |FC|. Each node in the network represents a protein, while its size indicates how many proteins it interacts with. The larger the node, the higher the number of proteins it interacts with. The protein’s expression level in the comparison group is indicated by the node’s color; orange denotes considerably high protein expression, whereas green denotes significantly low protein expression.

## 4. Discussion

The epididymis, seminal vesicles, prostate, and urethral bulbourethral glands are the primary sources of spermatozoa and secretions that make up an animal’s semen. Semen is made up of about 10% of sperm and 90% of seminal plasma. High levels of tissue-specific proteins found in seminal plasma thus offer a wealth and extremely promising source of indicators for evaluating male fertility. Sperm and seminal plasma proteins are among the proteins found in semen. They are directly engaged in controlling sperm function in molecular processes and biological pathways and significantly affect both semen viability and the fertilization capacity degree [6]. Semen proteins are, therefore, excellent potential indicators for assessing semen quality and checking for sperm dysfunction. The advancement of proteomics technology has enabled in-depth research on cellular mechanisms unique to sperm. These kinds of investigations are thus essential for finding and creating reliable biomarkers of male fertility. Previous studies postulate that seminal plasma and sperm proteins influence the ejaculatory process and the use of sperm biotechnology in fields such as artificial insemination, semen freezing, and sex sorting.

The management of reactive oxygen species (ROS) and energy supply are the two main facets of how mitochondrial proteins affect sperm quality. Mitochondria are “energy factories” inside the cell that supply the energy required for the cell to continue its regular functions. The entire intricate process of spermatogenesis to fertilization, which includes the vital step of accessing the egg, requires an enormous amount of energy. Mitochondria are thus essential and indispensable components in this process. Numerous studies postulate that the more active mitochondria are, the more energy they can create. Highly active mitochondria would thus further boost the quantity and health of spermatozoa and give them a stable energy source for their regular physiological functions. Of note, mitochondria are the primary source of reactive oxygen species (ROS). Maintaining the normal functional activities of spermatozoa, including sperm viability, fertilization ability, and important physiological processes such as energy acquisition, acrosome response, and hyperactivation, requires a suitable quantity of ROS. Oxidative stress is caused when ROS levels rise above normal, which damages sperm cells and causes them to gradually lose their ability to function. The damage to sperm cells ultimately has a detrimental effect on fertility. Studies postulate that between 30% and 80% of male infertility cases are caused by sperm destruction brought on by ROS [7,8]. The occurrence of lipid peroxidation in sperm cell membranes is closely associated with reduced sperm viability [9]. These reports collectively demonstrate that oxidative stress plays a significant role in sperm quality decline. Spermatozoa are highly polarized cells that generate energy internally to carry out various intricate and vital physiological functions, including energy acquisition, hyperactivation, and acrosome reaction. These functions are exactly what spermatozoa need to successfully complete fertilization [10]. Halang et al. reported that sperm viability is maintained using about 80% of the energy generated by spermatozoa through glycolysis and mitochondrial oxidative phosphorylation [11]. These reports explain the relevance of the differential expression of proteins in group B compared to group A in this experiment. Besides the impact of mitochondrial proteins, post-translational modification of sperm proteins also significantly affects sperm survival. Proteins can undergo post-translational modification when modifying groups are added or removed by covalently attaching to their amino acid residues. This phenomenon alters the structure and function of proteins and eventually impacts their activity [12]. Common post-translational changes in proteins include methylation, biotinylation, alkylation, phosphorylation, and acetylation. Post-translational modifications of proteins affect numerous critical physiological processes, including sperm maturation, capacitation, motility, hyperactivation, and acquisition of fertilization potential [13]. Mature spermatozoa rely on post-translational modifications of their existing proteins to maintain their normal physiological functions [14] because they synthesize a few new proteins [15,16,17,18] and undergo minimal gene transcription and translation, except the mitochondrial genes. The most prevalent post-translational alteration in spermatozoa is protein phosphorylation, which is essential for sperm maturation, energy uptake, and energy metabolism [19,20]. Sperm proteasome was initially discovered from sea squirt (Halocynthia roretzi). Notably, sea squirt exhibits trypsin-like and chymotrypsin-like activity together with 20S core particles during fertilization [21]. Sperms are prevented from accessing the yolk when high-affinity leupeptin analogs impede proteasome action. However, sperms may resume their usual ability to fertilize once this inhibitor is eliminated. This phenomenon implies that sperm capacitation and acrosomal cytotoxicity are significantly influenced by the activity of the proteasome’s β subunit [22,23]. The process of sperm capacitation potentially involves a mechanism that controls the entry of protein substrates into the proteasome’s core for eventual degradation because the ɑ subunit can control substrate entry and has been observed to undergo post-translational modification during capacitation [24]. This phenomenon also clarifies the importance of the proteins that are expressed differently in group B than in group C of this experiment.

The widespread neurodegenerative condition, Parkinson’s disease (PD) [25], may have a negative impact on the male reproductive system. Patients with Parkinson’s disease (PD) have several pathophysiological changes, including mitochondrial dysfunction and oxidative stress, which are the most common. Reactive oxygen species (ROS) are produced in significant quantities by oxidative stress, target lipids, proteins, and DNA in sperm cells, damaging the cell membranes and influencing sperm motility and morphology. Mitochondrial malfunction causes a decrease in sperm motility and fertilization because of inadequate sperm energy production [26]. According to neuroendocrine theory, PD patients may have disruptions in the hypothalamic–pituitary–gonadal (HPG) axis [27]. Neurotransmitter imbalances such as low dopamine levels cause the classic symptoms of Parkinson’s disease and affect pituitary gonadotropin production, which in turn influences the testes’ ability to produce sperm. Studies postulate that PD patients have lower testosterone levels, which directly inhibits spermatogenesis and maturation [28]. Moreover, the sperm may carry defective genes that potentially raise the offspring’s chance of developing Parkinson’s disease or other neurological illnesses. PD patients have lower-quality sperm, which can result in an irregular fertilization process and raise the risk of early problems in embryonic development, such as miscarriages, fetal developmental retardation, and unsuccessful embryo implantation [29]. Children borne of PD patients are at risk of neurodevelopmental abnormalities during growth and development, even in cases when conception and delivery are successful. Sheep with neurological disorders may exhibit altered sexual behavior, such as lower desire, which has an indirect impact on semen acquisition. Nonalcoholic fatty liver disease (NAFLD) is a prevalent liver illness [30] that causes metabolic problems, including insulin resistance and aberrant lipid metabolism, potentially impacting spermatogenesis negatively. Hormonal abnormalities caused by insulin resistance can impact the functioning of the HPG axis. Testicular spermatogenesis may be impacted by insulin resistance-induced changes in the insulin-like growth factor (IGF) system, including a reduction in IGF-1 levels [31]. Elevated blood levels of free fatty acids caused by abnormalities in lipid metabolism can penetrate testicular tissue and disrupt spermatogenesis and maturation. Patients with NAFLD have a diminished liver’s capacity for detoxification, which causes the buildup of toxins in the body, thereby negatively impacting the reproductive system [32]. The liver produces several cytokines and inflammatory mediators that enter the bloodstream and impact the testes’ milieu, causing spermatogenic cell damage, a drop in the number of viable sperm, an increase in the rate of malformation, and a decrease in viability. Sperm quality decline in males with NAFLD may have long-term consequences for the health of the offspring. Oxidative stress and metabolic disorders may damage and alter the sperm’s genetic material, which may then be passed on to the offspring, increasing the risk of metabolic diseases such as obesity and diabetes mellitus. Moreover, the paternal offspring’s NAFLD status may impact the embryo’s early development environment. Studies postulate that certain molecular signals in the sperm can transfer metabolic disorders in the paternal offspring to the embryo, altering the embryo’s gene expression and developmental trajectory. This phenomenon could potentially make the offspring more vulnerable to metabolic abnormalities as well as growth and developmental issues after birth. The aberrant metabolic environment of ewes with NAFLD may have an impact on the attachment and development of embryos. Metabolic disorders, such as hyperglycemia and hyperlipidemia, can harm the fetus and result in aberrant fetal development, including organ developmental anomalies. Alzheimer’s dementia (AD) is a complicated neurodegenerative illness whose pathological process disrupts the regular functioning of the neuroendocrine system. The brain levels of neurotransmitters, including dopamine and acetylcholine, are significantly altered in AD patients [33]. Neurotransmitters are essential for cognitive function and also play a role in controlling the activity of the hypothalamic-pituitary-gonadal (HPG) axis. The rhythm and quantity of gonadotropins released by the pituitary gland, such as follicle-stimulating hormone (FSH) and luteinizing hormone (LH), are also changed when hypothalamic production of gonadotropin-releasing hormone (GnRH) is impacted [34]. The regulation of spermatogenesis in the testicles is largely dependent on LH and FSH. Excess secretion disrupts the intratesticular environment of the testicles and interferes with spermatogonial proliferation, differentiation, and spermatogenesis, thereby affecting the amount and quality of sperm [35]. Sheep with neural cell injuries may also have reduced sexual behavior and reproductive function, which impacts the amount and quality of semen produced. The development of neuroprogenitor fibril tangles because of β-amyloid (Aβ) deposition and tau protein hyperphosphorylation is a primary pathogenic characteristic of AD. These aberrantly aggregated proteins impair the neuronal function in the brain and negatively affect the testicular tissue. The function of spermatogenesis-associated proteins is affected by Aβ and aberrantly phosphorylated tau proteins interfering with the normal folding, transport, and degradation activities of proteins in the testis. Several mutations in genes linked to AD, including those for amyloid precursor protein (APP), progerin 1 (PS1), and progerin 2 (PS2), have been reported [36]. Offspring of males with AD who inherit these faulty genes are at a significantly higher risk of getting AD or other neurological illnesses. Moreover, the pathophysiology of AD is significantly influenced by epigenetic changes, such as DNA methylation and histone alterations [37]. Sperm from AD patients may have changed epigenetic markers, which can be transferred to the progeny, which potentially impacts their gene expression pattern and increases their vulnerability to neurodevelopmental and functional problems. AD patients may experience aberrant fertilization because of a decline in sperm quality [38]. Damaged sperm may not be able to give the embryo the proper genetic and epigenetic support, which increases the risk of aberrant embryonic development, including early miscarriage and unsuccessful embryo implantation [39]. The genetic material and epigenetic information of the sperm are essential for early embryonic development. The baby may experience other neurodevelopmental issues despite the embryo growing properly, leading to neurodevelopmental issues such as cognitive dysfunction and diminished learning capacity. Sheep with Alzheimer’s disease may struggle to care for their offspring by neglecting to give them proper motherly attention, which might hinder the offspring’s growth and development. Moreover, the poor physical state of the sick sheep affects milk supply and quality, thereby negatively impacting the offspring’s growth and development. The phenomena of anomalies in ontogenetic performance, lambing %, and progeny quality in heterozygotes and purebreds have been discussed in the cited literature.

## 5. Conclusions

There are certain limitations due to the small sample size in each group. In this experiment, through proteomic analysis of semen from Duolang sheep rams with different FecB genotypes, the differentially expressed proteins (DEPs) in semen of the B+ genotype compared to the ++ genotype are significantly involved in the innate immune response. This indicates that these DEPs are significantly enriched in the oxidative phosphorylation pathway in the KEGG pathway, and exert effects on the immune health, energy metabolism, and reproductive performance of Duolang sheep. The DEPs in semen of the B+ genotype compared to the BB genotype play roles in protein phosphorylation and cellular protein catabolic processes. The pathways significantly enriched by these DEPs, including those related to Parkinson’s disease and non-alcoholic fatty liver disease, suggest impacts on the cellular metabolism, physiological functions, and reproduction of Duolang sheep. Additionally, differences in the molecular functions and cellular components of DEPs in semen of the BB genotype compared to the ++ genotype were analyzed, so as to explore the potential effects of these differences on the physiology and reproduction of Duolang sheep.

## Figures and Tables

**Table 1:** Differential protein screening.

Sample Pairs Comparing by the Former Over the Latter	The Number of Proteins Co-Identified in Both Sets of Samples	Regulated Type (Up- or Down-Regulation)	Up-Regulated Proteins (FC > 1.2 and *p* Value < 0.05), and Down-Regulated Proteins (FC < 0.83 and *p* Value < 0.05)
B.vs. A	2989	up-regulated	66
		down-regulated	35
C.vs. A	2827	up-regulated	81
		down-regulated	292
B.vs. C	2771	up-regulated	116
		down-regulated	28

**Table 2:** GSEA-KEGG enrichment results.

KEGG_ID	KEGG_Term	*p* Value	AdjustedPv	Size	ES	NES
MAP00190	OXIDATIVE PHOSPHORYLATION	0.0	0.019532768	97	−0.45213947	−2.0541892
MAP05012	PARKINSON’S DISEASE	0.0	0.0141317975	95	−0.45930517	−2.0012848
MAP04932	NON-ALCOHOLIC FATTY LIVER DISEASE _NAFLD_	0.0	0.045213815	70	−0.4325565	−1.8434372
MAP05010	ALZHEIMER’S DISEASE	0.0	0.07570649	98	−0.4006493	−1.7487847
MAP04723	retrograde endocannabinoid signaling	0.007633588	0.0965757	52	−0.42583093	−1.6883155

Note: (1) KEGG_ID: ID of the enriched KEGG Pathway; (2) KEGG_Term: name of the enriched KEGG Pathway; (3) *p* value: *p* value of the enrichment analysis. The *p* value is 0 when the actual *p* value is so small that it cannot be obtained accurately; (4) AdjustedPv: *p* value of the corrected (5) size: the number of proteins annotated to the functional entry; (6) ES: the enrichment score of the GSEA analysis of the entry; (7) NES: the enrichment score of the GSEA analysis of the entry after homogenization.

**Table 3:** GSEA-structural domain enrichment results.

IPR_ID	IPR_Term	*p* Value	AdjustedPv	Size	ES	NES
IPR000719	PROTEIN KINASE DOMAIN	0.0	0.046585634	46	−0.49194428	−1.8805562
IPR025933	BETA-DEFENSIN	0.006802721	0.02746326	17	−0.63333166	−1.8570249
IPR001353	PROTEASOME, SUBUNIT ALPHA_BETA	0.0033670033	0.019854257	17	−0.6215955	−1.8289802
IPR001623	DNAJ DOMAIN	0.01650165	0.0661915	16	−0.5654077	−1.6392351

Note: (1) IPR_ID: structural domain number; (2) IPR_Term: description of structural domain function; (3) *p* value: *p* value for enrichment analysis. The *p* value is 0 when the actual *p* value is extremely small and cannot be accurately obtained; (4) AdjustedPv: corrected *p* value; (5) size: the function entry annotated to the number of proteins; (6) ES: enrichment score of GSEA analysis for the entry; (7) NES: enrichment score of GSEA analysis for the entry after homogenization.

**Table 4:** GSEA-subcellular enrichment results.

Subcell_ID	*p* Value	AdjustedPv	Size	ES	NES
MITOCHONDRION PROTEIN	0.0	0.11314847	309	−0.2983893	−1.4824772
EXTRACELL PROTEIN	0.0	0.0	266	0.6574827	2.0779767
endoplasmic reticulum productin	0.024819028	0.1322063	120	0.4547453	1.3880157

Note: (1) Subcell_ID: description of subcellular localization; (2) *p* value: *p* value for enrichment analysis. The *p* value is 0 when the actual *p* value is very small and cannot be accurately obtained; (3) AdjustedPv: corrected *p* value; (4) size: the number of proteins that this functional entry annotates to; (5) ES: the entry GSEA analysis enrichment score; (6) NES: the enrichment score of the entry after homogenization by GSEA analysis.

## Data Availability

The original contributions presented in this study are included in the article. Further inquiries can be directed to the corresponding author.

## Abbreviations

AD	Alzheimer’s Disease
APP	Amyloid Precursor Protein
BP	Biological Process
CC	Cellular Component
COG	Cluster of Orthologous Groups
DIA	Data-Independent Acquisition
DEPs	Differentially Expressed Proteins
DTT	Dithiothreitol
FC	Fold Change
FSH	Follicle-Stimulating Hormone
GO	Gene Ontology
GnRH	Gonadotropin-Releasing Hormone
GSEA	Gene Set Enrichment Analysis
HPG	Hypothalamic-Pituitary-Gonadal
IGF	Insulin-like Growth Factor
IAM	Iodoacetamide
IPR	Inter Pro Scan
KEGG	Kyoto Encyclopedia of Genes and Genomes
LH	Luteinizing Hormone
MF	Molecular Function
NES	Normalized Enrichment Score
NAFLD	Nonalcoholic Fatty Liver Disease
PCA	Principal Component Analysis
PD	Parkinson’s Disease
PS1	Presenilin 1
PS2	Presenilin 2
ROS	Reactive Oxygen Species
SDS	Sodium Dodecyl Sulfate
SNAP-25	Synaptosomal Associated Protein 25
TEAB	Triethylammonium Bicarbonate
TFA	Trifluoroacetic Acid
UHPLC	Ultra-High-Performance Liquid Chromatography
